# Green Nanoparticles Engineering on Root-knot Nematode Infecting Eggplants and Their Effect on Plant DNA Modification

**DOI:** 10.15171/ijb.1309

**Published:** 2016-12

**Authors:** Kamal Fouad Abdellatif, Ragaa Hamouda Abdelfattah, Mostafa Sayed Mostafa El-Ansary

**Affiliations:** ^1^Department of Plant Biotechnology, Genetic Engineering and Biotechnology Research Institute (GEBRI), University of Sadat City, Sadat City, Egypt; ^2^Department of Microbial Biotechnology, Genetic Engineering and Biotechnology Research Institute (GEBRI), University of Sadat City, Sadat City, Egypt

**Keywords:** Eggplant, EST, Green Silver Nanoparticles, Molecular markers, Root-Knot Nematode, RAPD

## Abstract

**Background:**

Root-knot nematodes are known to cause significant damage to eggplants. New approaches by green silver nanoparticles (GSN) are used to control plant-parasitic nematode to avoid chemical nematicide hazards.

**Objectives:**

Analyses of the incorporation of different concentrations of nanoparticles on two different algae (*Ulva lactuca* and *Turbinaria turbinata*) were carried out. Furethermore, the effect of GSN on the eggplant DNA profile was studied using RAPD and EST molecular markers.

**Materials and Methods:**

Green Silver Nanoparticles (GSN) have been synthesized and characterized using the algal extract solution prepared from two algal genera. Nematicidal effect of the GSN was evaluated in greenhouse on eggplants (*Solanum melongena cv.* Login). Genomic DNA was extracted for use in molecular analysis. Both RAPD and EST molecular markers were used to study the GSN effect on eggplant DNA modification.

**Results:**

GSN (17 mg.mL^-1^) obtained from *U. lactuca* was more effective in reducing second-stage juveniles (J2s) of *M. javanica* (69.44%) population in soil. All treatments improved eggplants growth parameters. Change in DNA profile using of both RAPD and EST markers was noted.

**Conclusions:**

GSN (12.75 mg.100 mL^-1^) were effective on controlling the root-knot nematode (both *T. turbinata* and *U. lactuca* algae), similar to chemical control in eggplants. GSN did not cause any phototoxicity in eggplants under treatment.

## 1. Background

Eggplant’s growth can greatly be affected by rootknot nematode *Meloidogyne* spp. (1). Root-knot nematodes are categorized as important vegetable pests and their host range comprising more than 3000 plant species (2). Use of nematicides is the main management technique to lower the nematode impact. However, the nematicides such as Nemacur and Fenamiphos were considered to be unsafe and their use banned from 2008 (3). Biological control is another mean of suppressing the in filter atingnematods. However, such agents do have narrow host range and their potential is rather dependent on environmental conditions (4-5). Use of nanoparticles such as nanosilvers have been advocated in last few years to control plant pathogens including nematodes (6,7).

Genotoxicity, describes the property of chemical agents that damages the genetic information within a cell causing mutations,, induced by nanoparticles in plants is poorly understood (8). Although a few reports are indicative on DNA damage and genotoxicity in plants (9-10), some others such as use of silver nanoparticles on Bermuda grass report otherwise (11). Atha *et al*. (9) reported that nanoparticles constructed from copper oxide caused DNA damage in some plants (*Raphanus sativus*, *Lolium perenne* and *Lolium rigidum*). Rodriguez *et al* (10) stated that Comets, FCM-HPCV, and micronuclei are having almost the same effect of genotoxicity in plants.

Molecular marker techniques have been used to detect DNA damage and modification as a response to both biotic and abiotic stresses. Although Jo *et al*. (11) reported that silver nanoparticles did not cause any phototoxicity on Bermuda grass when they used it to control nematode infection; the molecular prove concerning DNA change still poorly understood. RAPD technique one of the methods that can be used to detect DNAdamage or modification and thus it could be used to study genotoxicity (12). Liu *et al.* (13) successfully used RAPD analysis to identify DNA damage and mutations in the Arabidopsis Cd-treated shoots. Expressed sequence tag (EST) is molecular marker that used in the evolutionary studies. It derived from transcribed regions of the genome so it could be considered more conserved than the other markers (14). EST markers have been used in many crop plants for those purposes (15,16). Up to date there is no report studying the DNA damage produced from application of nanoparticles on plants using EST markers.

## 2. Objectives

Green Silver Nanoparticles (GSN) have been synthesized and characterized using the algal extract solution prepared from two algae (*Ulva lactuca* and *Turbinaria turbinata*). This study aimed to evaluate the GSN application against root-knot nematode (*Meloidogyne javanica*) on eggplant under greenhouse conditions. Possibility of using GSN as plant fertilizers was also investigated. Furthermore, the effect of GSN on eggplant DNA was also seeked via molecular markers.

## 3. Materials and Methods

### 3.1. Synthesis of GSN

#### 3.1.1. Algal Extract Solution Preparation

*Ulva lactuca* and *Turbinaria turbinata* algal isolates were collected in May 2014 to be used for this purpose***, ***identified by Taylor (17). The marine alga *U. lactuca* was collected from Abu-Qir coast, Alexandria, Egypt. *T. turbinata* was collected from shallow water beside the shore of Red Sea, Safaga, Red Sea Governorate, Egypt. Algae samples were collected in polyethylene bags and were cleaned thoroughly by washing under running tap water to remove stones, epiphytes and extraneous matters. The alga were cut into small pieces and rinsed with sterile distilled water. The cleaned macro-alga was shade dried at room temperature for a week. The dried algae were powdered and aqueous extract was prepared by dissolving 1 g of the powder in 100 mL of distilled water and heated at 100ºC for 20 min. The extract was filtered and stored at **-**20ºC until use.

#### 3.1.2. GSN Solution Preparation

AgNO_3_ (17 mg) was slowly dissolved in 100 mL of the previously prepared algal extract solution using magnetic stirrer for even coating of silver and subjected to heating at 55°C for 10 min for reduction of GSN. The reduction was monitored by color changing from bale yellow to red. Furthermore, bioreduction was also measured via UV-vis spectra at wavelength of 100-700 nm on UV-Visible spectroscopy (T80+UV/VIS Spectrometer).

#### 3.1.3. Analysis of GSNs

Silver nanoparticles synthesized by *T. turbinate* were analyzed using X-Ray diffractometer (XRD, type JED-2300T). The Cu-Kα X-rays of wavelength 1.54060 Å was obtained and data were taken for 2θ at range of 10° to 80° with a step of 0.026°. X-Ray Generator operated at a voltage of 45kv and current of 30mA with Cu-Kα radiation. Estimation of the size, shape and state of assembly of the GSN were conducted using Transmission Electron Microscopic (JEOL JEM-2100). Energy -dispersive X-ray (EDX) was carried out using JXA-840A instrument.

### 3.2. Nematicidal Evaluation

#### 3.2.1. Pure Nematode Culture

Eggs of nematode (*Meloidogyne javanica*) were extracted from infected tomato (cv. Castle Rock) roots;using sodium hypochlorite solution according to (18). Second-stage juveniles (J2s) of nematode were collected daily from eggs and were stored at 15°C. The juveniles used in the experiments were less than 5 days old.

#### 3.2.2. Greenhouse Assays

Eggplants (*Solanum melongena*) were cultivated in plastic pots to study the management of root-knot nematode by applications of silver nanoparticles. One month old eggplants (cv. Login) were planted in 30 cm diameter plastic pots containing a mixture of 1:2 sterilize clay/sand soil. Pots (40) were inoculated with 1,000 (J2s) per pot at the planting time. Six days later, 32 pots were treated with GSNs, obtained from two types of marine algae as following (four pots to each treatment representing four replicates): using the alga *T. turbinate* A: 17 mg.100 mL^-1^, B: 12.75 mg.100 mL^-1^, C: 8.5 mg.100 mL^-1^, D: 4.25 mg.100 mL^-1^, using the alga *U. lactuca* E: 17 mg.100 mL^-1^, F: 12.75 mg.100 mL^-1^, G:8.5 mg.100 mL^-1^, H: 4.25 mg.100 mL^-1^. Four inoculated pots were treated using chemical control (2 mL Vaydte®/pot), Oxamyl {Vydate® L24% Methyl-N, Ndinethyl N-(methyl carbamayl) oxythioxamidate}. The remaining four inoculated pots were served as infected without treatment application. One treatment without application was kept as control (control without nematode). The treatments were added to the soil in 3 holes made in soil. The algal extracts were used in very low concentrations for reduction of the nanoparticles.

#### 3.2.3. Data Collection and Statistical Analysis

The treatments were distributed in a completely randomized design in a greenhouse of Genetic Engineering Biotechnology Research Institute (GEBRI), University of Sadat City, Sadat City, Minou fiya, Egypt. Plants were evaluated after 45 days of inoculation. The roots were washed carefully to remove soil and stained with Phloxine B solution for 5 min to facilitate counting of egg-masses. Nematode reproduction parameters were number of galls, females, egg-masses per root system and number of juveniles per 250 gr soil. The plant growth parameters were shoot length (cm), shoot weight (g), root length (cm), root weight (g) and number of leaves. The responses of the treatments were compared by analysis of variance according to Sokal and Rohlf (19). Significant differences among the means of parameters were determined by using Duncan’s multiple range tests (P≤0.05). All analyses were carried out using with SPSS software.

### 3.3. Molecular Characterization

#### 3.3.1. DNA Extraction

Young leaves of GSN treated eggplants next to controls were ground to powder in a mortar with liquid nitrogen. DNA was isolated from 60 mg of the ground leaves tissues using i-genomic plant DNA extraction mini kit (INTRON Biotechnology, Inc.) according to the manufacturer instruction. DNA concentration was adjusted at 25 mg.μL^-1^.

#### 3.3.2. Molecular Markers Analysis

Six random 10-mer primers were used for RAPD analysis (Table 1). A total volume of 25 μL PCR reaction was used for PCR analysis containing 75 ng DNA template, 200 μM dNTPs, 1.5 mM MgCl2 , 5 μL of 5×Taq polymerase buffer, 0.25 μM of the primer and 0.75 U *Taq* DNA polymerase. The PCR cycling condition involved initial denaturation at 94ºC for 5 min. followed by 35 cycles of 94ºC for 40 sseconds, 32ºC for 35 seconds and 72ºC for 40 seconds and a final 72ºC for 4 minutes.

**Table 1:** EST and RAPD primer sequences to detect DNA change in eggplant

**EST Primer**	**Sequence (3'-5')**	**RAPD Primer**	**Sequence (3'-5')**
FC15 FC22 FC27 FP21 FP22 FP25	F: CTCATCCCTTGCTTACCTTA R: CCAAATTGCACTTGAAATAA F: GATTTCAGAGGTCATTCCAA R: CAAACTACATGGATCAAGCA F: CTGGTCATGTGGGAAGTAGT R: ATAAATGTGGAAGGCTCAAA F: AGACGAACCAGAAGACGTTA R: ATATGAACCAGCTAGGCAGT F: AGAATGGACTTTGAAGCTGA R: CGAAATAGGAGACGAAGTTG F: GAAGCGTCACATTTAACTCC R: ACAAATTCTGAATGCATGAC	OPA07 OPA09 OPB10 OPB12 OPC05 OPR02	GAAACGGGTG GGGTAACGCC CTGCTGGGAC GTAGACCCGT GATGACCGCC CACAGCTGCC

EST primers (6 pairs) were used to perform the molecular differentiation (Table 1) according to (20). The PCR amplification reactions were achieved in a 25 μL volume using 50 ng DNA containing 0.25 μM of each primer, 250 μM of dNTPs, 5 μL of 5×*Taq* polymerase buffer, 1.5 mM MgCl2 and 0.75 U *Taq* DNA. The EST reactions were carried out using Touchdown PCR:7 cycles at 94ºC:45 seconds, 52ºC:45 seconds, decreasing 1ºC in every cycle, and 72ºC for 50 seconds, followed by 28 cycles at 94ºC: 45 seconds, 46ºC:45 seconds and 72ºC:50 seconds. The previous cycles were preceded by a denaturation step at 94ºC for 5 min and ended by 72ºC for 5 min.

#### 3.3.3. Data Handling and Cluster Analysis

All PCR products were separated on 1.5% agarose gel electrophoresis and the gels were scored for computer analysis on the basis of the presence of the amplified products for each primer. The bulked EST and RAPD data were used to generate a dendrogram. NTSYS-pc software was used to determine similarity coefficient matrices using simple matching imilarity algorithm (21). The similarity coefficient was used to construct dendrogram using UPGMA in order to analyze genetic similarities (22). Dendrogram was used to determine the genetic similarities among the different genetic materials under study.

## 4. Results

### 4.1. Characterization of Silver Nanoparticles

**Figure 1:**
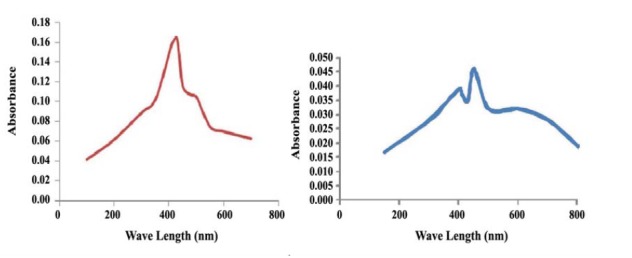
UV-Vis absorption spectra of green nanoparticles (GSN) synthesized from *T. turbinata *(left) and *U. lactuca* (right) extracts by treating with 1 mM silver nitrate

The green silver nanoparticles (GSN) were characterized by UV-Vis spectroscopy. A single broad peak was observed at 434 nm, corresponding to plasmonex citation, for GSN synthesized by the algae *Turbinaria turbinata* and *Ulva lactuca* (Figure 1). The efficiency of a ccomodation of the GSN by *T. turbinata* (0.16) was higher than *U. lactuca* (0.045). Accordingly, XRD and EDX experiments were carried out only on *T.turbinata*.

The diffracted intensities from 10° to 80° at 2 theta angles degrees were 27.66°, 32.10°, 46.0°, 54.826°, 57.484°, 67.462°, 74.473°, 76.750°, which can be indexed to (110), (111), (200), (220), (311), (222), (400) (331 and (420) sets of lattice planes, respectively.

**Figure 2:**
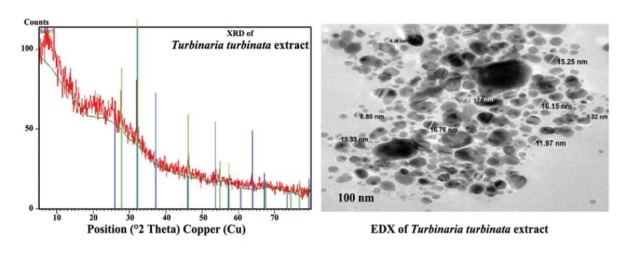
XRD pattern (left) and TEM images (right) of GSN synthesized from *T. turbinata* extract, treated by 1 mM silver nitrate

The data illustrated that silver ions were reducted by the algal extract of *T.turbinata*. The X-ray diffraction showed two intense peaks at 27.94 and 32.27, which corresponds to (110) and (111) of Ag_2_O (Figure 2).

TEM micrograph recorded from the GSN deposited on carbon coated copper TEM grid showed spherical particles in the range of 8-19 nm (Figure 2). EDX was used to verify the presence of silver in the suspension of nanoparticles. The vertical axis displays the number of x-ray counts whilst the horizontal axis displays energy.

**Figure 3:**
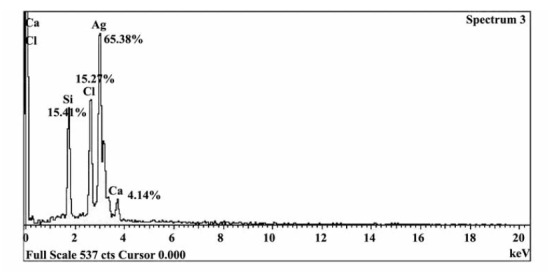
EDX spectrum and percentage of elements in suspension of GSN synthesized from *T. turbinata* extract, treated with 1 mM silver nitrate

Quantitative analysis proved high silver contents (65.38%) in the samples synthesized by *T. turbinata*. Other elements showed different values; for instance Si, Cl and Ca were 15.41%, 15.27% and 4.14%, respectively (Figure 3).

### 4.2. Evaluation of Antinematicidal Effect

Influence of silver nanoparticles (GSN) on development of nematodes on eggplants was evaluated.

*Meloidogyne javanica* reproduction and growth were affected. All treatments were comparable (K) to chemical control (Vydate®24%L); resulted in significant reduction in number of galls, number of female nematodes, and number of egg-masses per root system (Table 2). The same finding was noticed on the number of nematode juveniles tow (J2s) in soil compared with those of (M) control (infected plants).

**Table 2:** Efficacy of GSN on reproduction parameters of *M. javanica* in eggplant

** Treatments**	** (mg.100 mL^-1^) **	** Galls/ root **	**Females/ root**	** Egg masses/ root **	**Juveniles (J2s)/ 250 soil**
*Turbinariaturbinata* *Ulva lactuca* Chemical control Control (Nematode) Control	A (17) B (12.75) C (8.5) D (4.25) E (17) F (12.75) G (8.5) H (4.25) K(2 ml Vydate®) L (Infected) M (uninfected)	53 ed 99cd 95.75bcd 96.25bcd 51e 62.75edc 86.5bcde 114b 45.75e 438 a -	46.25b 55b 67.25b 106.25b 54b 65.25b 57.25b 73.5b 47.5b 298.5a -	58.25b 74.5b 72b 130.25b 58.5b 72.5b 91b 111b 57.5b 278.5 a -	1116c 1140c 1308c 1894b 1023c 1127.5c 1219c 1916b 948c 3348a -

Means connected with the same letter(s) within a column are not significantly different (P ≤ 0.05) according to Duncan’s Multiple Range tests.

**Figure 4:**
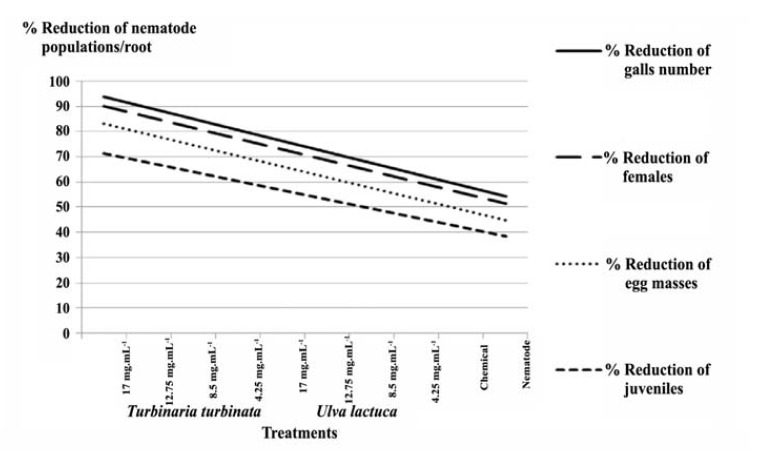
Reduction percentage of nematode population parameters after treatment with GSNsynthesized by marine algae on eggplants and its comparison with cases treated with nemato- cide was implemented and the untreated plants (infected)

Generally, the concentration of 17 mg.100 mL^-1^ (treatment E) of *U. lactuca* with GSN was the most effective treatment in reduction of *M. javanica* population (69.44%) J2s in soil. Asimilar effect was noted for chemical control (K, 70.61%). The nanoparticles produced by *T. turbinata* (A) reduced the number of females in roots by 84.51% that was comparable with (K) (84.09%) (Table 2 and Figure 4).

**Figure 5:**
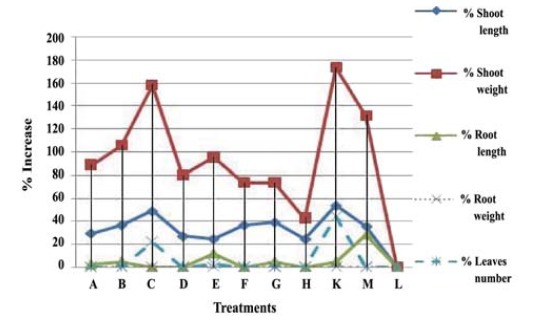
Percentage of growth increase in eggplant due to application of GSN in the presence and absence of *M. javanica*. A-H refer to treatments of different concentrations of nanoparticles, K refers to (2 mL Vydate/pot), L refers to the infected control and M refers to the uninfected control

Growth parameters of eggplants, weight and length of shoots and roots and number of leaves, are listed in Table 3. All treatments showed improvement in plant growth. However, no remarkable increase in root weight parameters was noticed (Table 3 and Figure 5).

**Table 3:** Growth reactions of eggplant treated with application of GSN synthesized by marine algae, and chemical nematocides in the presence and absence of root-knot nematode

** Treatments**	** (mg.100 mL^-1^) **	** Shoot **		** Root **		**Leave**	
		** length (cm) **	**weight (g) **	**length (cm) **	**Weight(g) **	**Number**
*Turbinaria turbinata* *Ulva lactuca* Chemical control Control (Nematode) Control	A (17) B (12.75) C (8.5) D (4.25) E (17) F (12.75) G (8.5) H (4.25) K(2 mL Vydate®) L (Infected) M (uninfected)	26.5abc 28 bcd 30.5 cd 26 ab 25.5ab 28bcd 28.25abc 25.5ab 31.5 d 20.5 a 27.75 abcd	10.79bcd 11.73cd 14.77e 10.24bc 11.17cd 9.92bc 9.91bc 8.16b 15.66 e 5.72 a 13.26 de	10.75a 11ab 10a 9.75a 11.75 a 9.5a 11ab 9a 11ab 10.5a 13.5 b	3.16ab 5.17ab 4.19ab 4.08ab 4.05ab 2.87ab 3.7ab 2.93ab 3.81ab 6.08a 1.58b	8ab 7.75ab 9.75bc 7.75ab 25.5ab 28ab 28.25ab 25.5a 11.5c 8ab 6 a

Means connected with the same letter(s) within a column are not significantly different (P ≤ 0.05) according to Duncan’s Multiple Range tests.

### 4.3. Analysis of Molecular Pattern

**Figure 6:**
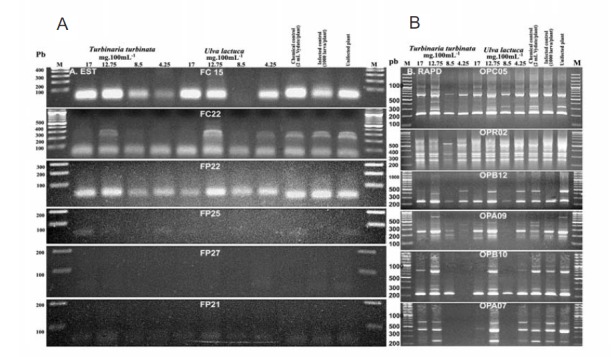
DNA pattern of eggplant treated with nanoparticles samples, (A) EST molecular markers and (B) RAPD markers. GeneRuler 100 pb DNA ladder (Thermo Scientific) was used to indicate the fragments size

The amplicon sizes of EST molecular patterns were 200-300 bp. The number of amplified fragments per primer pair ranged from one fragment (FP21 and FP27 primer pairs) to three (for the primer pair FC22). The pattern of the three controls (e.g. uninfected plant, infected plant and the chemical control) seems to be almost the same according to EST pattern (Figure 6A). For RAPD pattern, the rangees of amplified fragments were between 150-800 bp. The more related patterns are the three controls.

### 4.4. Analysis of Genetic Similarity Using Molecular Markers

**Figure 7:**
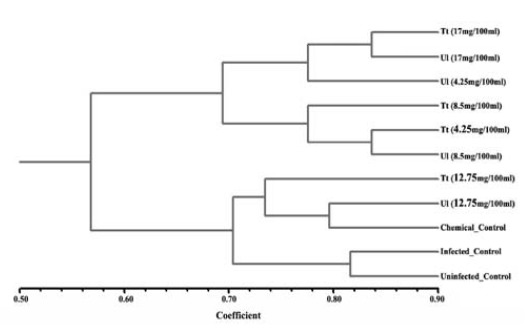
Dendrogram of eggplant DNA damage caused by nanoparticles and/or chemical treatment in the presence/absence of *M. javanica* generated from RAPD and EST molecular markers

The data from both RAPD and EST analyses were bulked in one file and used to produce a dendrogram using UPGMA algorithm. The samples were divided into two main groups. The first group containing the uninfected plant along with the infected plant (separated together in the lower most of the dendrogram) in addition to the samples obtained from the chemical treated plant, the concentration of 12.75 mg.100 mL^-1^ of silver nanoparticles incorporated in both *T. turbinate* and *U. lactuca* algae. The other group (in the upper most of the dendrogram) included the other treated samples (concentrations of 17, 8.5 and 4.25 mg.100 mL^-1^ of silver nanoparticles incorporated in both *T. turbinata* and *U. lactuca* algae (Figure 7).

It seems from this analysis that all treatments affected the genetic material of eggplant (whether the infected plant with nematode; chemical control; or silver nanoparticles application against root-knot nematode).

Infected plant has the lowest effect on DNA change (the nematode infected plant was clustered with uninfected plant and they were the most related samples). The effects of chemical control as well as 12.75 mg.100 mL^-1^ concentration of silver nanoparticles incorporated in both algae were similar on DNA change and lower than other concentrations. Furthermore, nanoparticles obtained from *U. lactuca* produced less DNA change compared with nanoparticles from *T. turbinata*.

## 5. Discussion

This study was carried out to investigate the effect of green silver nanoparticles (GSN) as nematicide against root-knot nematode (*Meloidogyne javanica*) infecting the eggplant. Moreover, the effect of GSN on DNA change of eggplants was studied using different molecular markers (*i.e*. RAPD and EST).

The GSN were characterized using UV-Viz Spectroscopy at wavelength of 434 nm. Several researchers have observed absorption maxima of colloidal silver solution at wavelength between 410 to 440 nm, which is assigned to surface plasmon of various metal nanoparticles (23). The fluorescence spectrum showed a broad emission peak of GSN at 414 nm when excited at 432 nm, Similar to what was reported by (24).

These peaks corroborate with the standard Ag_2_O (25). A number of strong Bragg reflections were identified corresponding to (111), (200), (220), (311) sets of lattice planes, which was agreed with that observed previously (26). Peaks of 57.484°, 67.462°, 74.473° and 76.750° were observed and they were corresponded to AgCl. TEM micrograph of *T. terbinata* showed spherical GSN in the range of 8-19 nm in size, smaller than the sizes reported (20-56 nm) for *U. lactuca* (27).

Root-knot nematodes (*Meloidogyne* spp.) are most important and very obligatory group of plant-parasitic nematodes occurring all over the world but found more in areas having worm climate. Natural management of nematode has become a feasible alternative to the nematicides. Here, different concentrations of GSN were used for management of root-knot nematode through two types of algal extracts. Inhibition of *M. javanica* (J2s) through soil inoculums, demonstrated strong nematicidal effects of algae with GSN.

Greenhouse evaluation of GSN, biosynthesized in algae demonstrated its beneficial effect for root-knot nematode management and root gall reduction at all stages of nematode life cycle. The GSN are reduced by the algal extraction. All treatments of GSN became more efficient to root-knot nematode management because of their protection from additional feebleness and stress by their incorporation on the algal extracts.

The nematicidal effect of algae with GSN was tested against root-knot nematode (*M. javanica*) compared with previous toxicological studies based on non parasitic nematode, *Caenorhabditis elegans* (28,29). The nematicidal effect was further confirmed by application of GSN in soil (17,12.75,8.5 and 4.25 mg.100mL^-1^). The data was demonstrated efficient reduction of J2s in soil. It can be noticed also that all concentrations of tested materials as well as chemical control (Vydate®24%L) caused a remarkable increase in plant growth parameters as opposed to controls. Such notion might be due tomarine algae, which contains all major and minor nutrients and many organic compounds such as auxins, gibbrellins, and precursor of ethylene and betaine (30,31). Thus, it can be said that mode of action of GSN is not specific and associated with disrupting multiple cellular mechanisms including membrane permeability, ATP synthesis and response to oxidative stress in both eukaryotic (6,28) and prokaryotic cells (32). So, combining GSN with algae such as *T. turbinata* and *U. lactuca* that supplement the GSN nematicidal effect may increase its applicability. Additionally, understanding of the mechanism in the nematicidal action of GSN also permits improvement of GSN effectiveness by addition natural compound (*i.e*. algae formulations).

Eggplant DNA change after application of GSN has been studied using two different types of molecular markers. A dominant marker (RAPD) was chosen to monitor the changes at the random level, while a specific co-dominant marker (EST marker) was selected to illustrate if any changes in specific regions can be determined. According to this study it can be concluded that infection of eggplants with root-knot nematode causes DNA change as well as using of chemical treatments to suppress the root-knot nematode. The same results have been noted from application of GSN to manage nematode infection, but with different degrees. The lowest effect of nanoparticles on DNA change could be obtained from using concentration of 12.75 mg.100 mL^-1^ incorporated on the alga *U. lactuca*. The lower concentrations caused more DNA changes according to the molecular analysis. Aggregation of GSN in the soil may be accompanied with the higher concentration preventing their penetration inside the plant cell and consequently decreasing their effect on DNA change. The same results were found in the literature (33). They studied effect of zinc peroxide (ZnO_2_) nanoparticles on DNA and protein integrity on human peripheral blood mononuclear cells. They reported that ZnO_2_ nanoparticles at a minimum concentration of 5 μg.mL^-1^ were capable of promoting aggregation of malate dehydrogenase, and facilitated its degradation at higher concentrations. The changes in DNA resulted from application of 12.75 mg.100 mL^-1^ incorporated on the alga *U. lactuca* is comparable to those resulted from application of chemical treatments. According to literature, GSN did not cause any phototoxicity on bermudagrass, when they used to control nematode infection (11). Thus it can be said that application of 12.75 mg.100 mL^-1^ incorporated on the alga *U. lactuca* as well as application of chemical control cause the lowest DNA change on eggplants when it was used as nematicides. Although silver nanoparticles cause DNA change, their level still may be in the safe side because they were clustered along with the uninfected plant according to molecular cluster analysis. In general, *U. lactuca* caused less eggplant DNA change than *T. turbinata*. According to Figure 1, absorbance of GSN in *T. turbinata* was about 3.5 times of its absorbance in *U. lactuca*. This means that the amount of GSN in *U. lactuca* extract was less than its amount in *T. turbinate* extract and consequently, could explain why it has less effect on DNA change of the eggplants. Furthermore, the phytochemical compounds in *U. lactuca* are different from those in *T. turbinata*, which may be the reason why we have seen different effect on plant cell viability and its content (27).

## 6. Conclusions

Greenhouse assays attested to the nematicidal effect of green nanosilver for managing *Meloidogyne javanica* infecting eggplants*.* So, incorporation of silver nanoparticles on the algae has been proven by XRD, EDX and TEM micrograph analyses. The quantitative analysis also proved high silver contents (65.38%) in the samples synthesized by *T. turbinata*. Noteworthy, all treatments of GSN became more efficient to root-knot nematode management because of their protection from additional feebleness and stress by their incorporation on the algal extracts. Also, all concentrations of treatments caused a remarkable increase in plant growth. Considering DNA modification, it can be concluded that all tested materials causes DNA change in eggplant, but with different degree. This study supplied evidence that, green GSN may have beneficial current pesticides for controlling rootknot nematode to avoid chemical nematicides hazards.

## Acknowledgments

The authors are grateful to Mr. Mohamed E. Abdel- Aal for his help during this work, Plant Biotechnology Dept., (GEBRI), University of Sadat City, Egypt.
